# Effects of Current Output Modes on Corrosion Resistance of Micro-Arc Oxidation Black Coatings on Aluminum Alloy

**DOI:** 10.3390/ma18132949

**Published:** 2025-06-22

**Authors:** Shiquan Zhou, Rui Tong, Hongtao Li, Xiang Tao, Jian Chen

**Affiliations:** College of Materials Science and Engineering, Nanjing Tech University, Nanjing 211816, China; 202261203164@njtech.edu.cn (S.Z.); 202261203248@njtech.edu.cn (R.T.); 202261203226@njtech.edu.cn (X.T.); 202361203196@njtech.edu.cn (J.C.)

**Keywords:** micro-arc oxidation, current mode, energy consumption, corrosion resistance

## Abstract

In this work, micro-arc oxidation (MAO) under constant- and gradient-current modes was used to modify the surface of 6061 aluminum alloy. A black coating was created in situ on the alloy surface by controlling the spark discharge parameters during MAO. Using an electrochemical workstation (Metrohm Autolab, PGSTAT302 N, Herisau, Switzerland), energy-dispersive spectroscopy (EDS, JEOL, JSM-IT500A, Tokyo Metropolis, Japan), and scanning electron microscopy (SEM, JEOL, JSM-7900F, Tokyo Metropolis, Japan), the effects of the current output modes on the coating growth rate, energy consumption, colorimetric parameters (L*, a*, b*), microstructure, and corrosion resistance were methodically examined. The findings showed that the gradient-current mode (6 → 4 → 2 A/dm^2^) greatly lowered the micropore size (from 3.89 μm to 1.52 μm) and improved the coating compactness (porosity dropped by 40%), and all coatings satisfied the necessary blackness criterion (L* < 30). Additionally, this mode achieved excellent corrosion resistance, as demonstrated by a one-order-of-magnitude reduction in the corrosion current density (2.55 × 10^−8^ A/cm^2^ vs. 2.34 × 10^−7^ A/cm^2^), while minimizing the energy consumption (2.37 kW·h/m^2^·μm vs. 3.45 kW·h/m^2^·μm for constant current).

## 1. Introduction

As a lightweight structural material, aluminum alloy offers a wide variety of application opportunities [1,2]. The 6061 aluminum alloy is one of the most widely used and produced aluminum alloys in the world. It is characterized by its excellent cost-effectiveness, good overall mechanical properties, and application-matching properties. These properties, among other aspects, make it a popular choice in a variety of fields, including aerospace, precision machinery, and electronic equipment housings. Consequently, the micro-arc oxidation process for 6061 aluminum alloy is also highly mature and optimized, and it is relatively straightforward and reliable to obtain high-quality coatings on its surface. However, due to poor corrosion resistance under particular circumstances, its practical usefulness is severely limited [3,4]. One eco-friendly and sustainable surface treatment method is micro-arc oxidation [5]. A high voltage is applied to the aluminum alloy’s surface to create a micro-arc discharge during the micro-arc oxidation process. The substrate’s passivation layer is broken down by the high-temperature and high-pressure conditions created during this process, and the substrate’s aluminum and oxygen mix to produce a ceramic oxide coating [6,7,8]. The mechanical and corrosion resistance of aluminum alloys may be significantly enhanced by this coating, which typically has excellent hardness, wear resistance, and corrosion resistance [9,10,11].

In the field of optical instruments and 3C products, aluminum alloys are mainly used for black products, especially to satisfy light absorption requirements. For example, to reduce the impact of light on measurement results, the market requires the black value of the shell to be less than 30 (the L* value represents the brightness of an object, with 0–100 representing the range from black to white). In other words, one way to judge the black color is to have an L* value of less than 30.

Several studies have shown that four crucial criteria primarily affect how well micro-arc oxidation coatings work on aluminum alloys: the electrolyte composition (the primary determinant of coloration [12,13]) and concentration [14,15], the electrical parameters (including the voltage, current density, duty cycle, frequency, and oxidation time) [16,17], additives [18], and the substrate composition [19]. At the same time, a lot of research has been performed to clarify the principles of coloring and the development mechanisms of MAO coatings [20,21]. Nonetheless, prior research has recognized intrinsic constraints linked to the plasma discharge properties during MAO processing, including the development of discharge channels and microcracks in the ceramic coatings. The total corrosion resistance of the coatings is compromised by these structural flaws, which act as preferred entry points for corrosive media [21,22].

Two main approaches to improving the coating performance through microstructural alteration have emerged in response to this technical challenge. First, the plasma discharge characteristics have been successfully controlled by process parameter optimization, which involves fine-tuning the electrical parameters, soft sparking conditions, and electrolyte composition. This method improves coating densification and defect reduction by allowing for the adjustment of the arc discharge energy and size [23]. Second, the creation of composite coatings by combining organic additives, corrosion inhibitors, and nanoparticles has shown encouraging outcomes. During MAO processing, these functional additives are propelled by electric field forces to migrate preferentially to defect sites, such as micropores and microcracks, resulting in efficient pore sealing [24] and improved corrosion resistance [25,26,27,28].

The synergistic management of the current density, duty cycle, and frequency, either alone or in combination, has been the main focus of existing research on electrical parameter optimization (the first technique) [29,30]. Interestingly, research on gradient parameter modifications (such as progressive current density, duty cycle, and frequency fluctuations) is still rather limited. Moreover, research on the optimal timing for the adjustment of the electrical parameters is predicated on a simple time-based division, i.e., the oxidation duration is fixed and the output parameters are adjusted concurrently [31]. As is well established, the micro-arc oxidation process can be subdivided into three distinct phases of discharge. The form of discharge is determined by the output energy. An increase in both the oxidation duration and coating thickness necessitates a higher energy output to penetrate the coating and thus promote continued growth. However, an excess of energy can also result in damage to the coating. The purpose of the gradient output mode is to modify the discharge form by adjusting the output energy. It is evident that the straightforward oxidation time, utilized to determine the adjustment of the output parameters, is inadequate to achieve precise control over the discharge process. Specifically, when adjusting the output parameters, micro-arc oxidation is conducted to its midpoint and late stages. Consequently, this approach is not particularly meaningful when attempting to adjust the output parameters for the destructive discharge process. The micro-arc oxidation process, using different forms of discharge, exhibits divergent characteristics, including the color of the spark and the volume of the popping sound. These variations in characteristics are discernible to the naked eye, facilitating the observation of the transformation in the discharge form. This work presents a novel current regulation procedure based on real-time plasma discharge monitoring in order to fill these information gaps. In contrast to traditional time-scheduled methods, this technique uses real-time current density changes that are prompted by transitory changes in the properties of spark discharge during MAO processing. The existing output modalities (continuous regulation versus stepwise adjustment) and their effects on the coating growth kinetics and architectural development may now be fundamentally investigated thanks to this paradigm change.

## 2. Materials and Methods

### 2.1. Sample Preparation

Cylindrical specimens (35 mm in diameter × 3 mm thickness) were machined from 6061 aluminum alloy rods with the following nominal composition (wt.%): 0.8–1.2 Mg, 0.4–0.8 Si, ≤0.7 Fe, 0.15–0.4 Cu, ≤0.25 Zn, 0.04–0.35 Cr, ≤0.15 Ti, ≤0.15 Mn, and balanced Al. Prior to surface treatment, all specimens were progressively ground with silicon carbide (SiC) abrasive papers of up to 2000 grit to achieve an average surface roughness (Ra) of 0.1 ± 0.02 μm. Subsequently, the samples underwent ultrasonic cleaning in acetone for 5 min to remove organic contaminants, followed by thorough rinsing with deionized water and drying in a nitrogen atmosphere.

### 2.2. Micro-Arc Oxidation Treatment

The coatings were fabricated using a unipolar pulse power(JCL, JCL-21KW, Chengdu, China) supply under two distinct current modes: constant current densities (2, 4, and 6 A/dm^2^) and programmed gradient-current modes (2 → 4 → 6 A/dm^2^, hereafter designated as “246” mode; 6 → 4 → 2 A/dm^2^, hereafter designated as “642” mode), with a fixed processing duration of 15 min. During MAO treatment, the aluminum alloy specimen served as the anode, while a stainless-steel cylindrical counter-electrode (cathode) was concentrically arranged with a 8.5 cm interelectrode gap. The electrolyte composition comprised 30 g L^−1^ sodium hexametaphosphate ((NaPO_3_)_6_), 5 g L^−1^ sodium silicate pentahydrate (Na_2_SiO_3_·5H_2_O), and 5 g L^−1^ ammonium metavanadate (NH_4_VO_3_), with its temperature maintained below 30 °C using a recirculating chiller system. The electrical parameters were controlled at a duty cycle of 20% and frequency of 500 Hz. Post-treatment, the coated specimens were sequentially rinsed with deionized water, subjected to multi-cycle ultrasonic cleaning (3 × 5 min), and immediately dried under a compressed air flow to prevent hydration reactions.

### 2.3. Coating Characterization

Coating thickness measurements were performed using a handheld eddy current thickness gauge (Helmut Fischer GMBH, FMP20, Sindelfingen, Germany) with ±0.5 μm measurement accuracy. Surface darkness quantification was conducted through L*a*b* color space analysis employing a calibrated colorimeter (Linshang, LS171,Shenzhen, China).

Microstructural characterization was carried out via a multi-scale microscopy approach: primary morphological analysis using scanning electron microscopy (SEM, JEOL, JSM-7900F, Tokyo Metropolis, Japan) at a 15 kV accelerating voltage; high-resolution cross-sectional imaging and elemental mapping performed by field-emission SEM (FE-SEM, JEOL, JSM-6700F, Tokyo Metropolis, Japan) coupled with energy-dispersive X-ray spectroscopy (EDS, JEOL, JSM-IT500A, Tokyo Metropolis, Japan). Quantitative analysis of the pore characteristics (size distribution and areal porosity) was achieved through the threshold segmentation of SEM micrographs using the ImageJ(ImageJ-win64) software, with a minimum of 10-image sampling per condition. Dielectric strength evaluation was implemented via dielectric breakdown testing (Junhui, ZC7122D, Shenzhen, China). The voltage was ramped at 100 V/s until the leakage current exceeded 10 mA, with the breakdown voltage recorded as the potential difference at insulation failure. Results were normalized as the dielectric strength (kV/mm) using the measured coating thickness.

The corrosion resistance of MAO coatings was evaluated using a Metrohm Autolab PGSTAT302 N electrochemical workstation(Herisau, Switzerland). A conventional three-electrode cell system was applied, in which a sample with a surface area of 1 cm^2^ was used as the working electrode, a saturated glycerol solution as the reference electrode, and a platinum foil as the counter-electrode. Electrochemical measurements were conducted in a 3.5 wt.% NaCl solution maintained at 25 ± 1 °C using a thermostatic cell. Prior to potentiodynamic polarization measurements, the system was stabilized at the open-circuit potential (OCP) for 60 min to establish steady-state conditions. Polarization curves were acquired with a scan rate of 1 mV/s over the potential range from −0.5 V to +0.5 V relative to the OCP. All tests were performed in triplicate under quiescent electrolyte conditions using freshly prepared solutions.

## 3. Results and Discussion

### 3.1. Effects of Current Output Modes on Growth of Micro-Arc Oxidation Coatings

**Figure 1:**
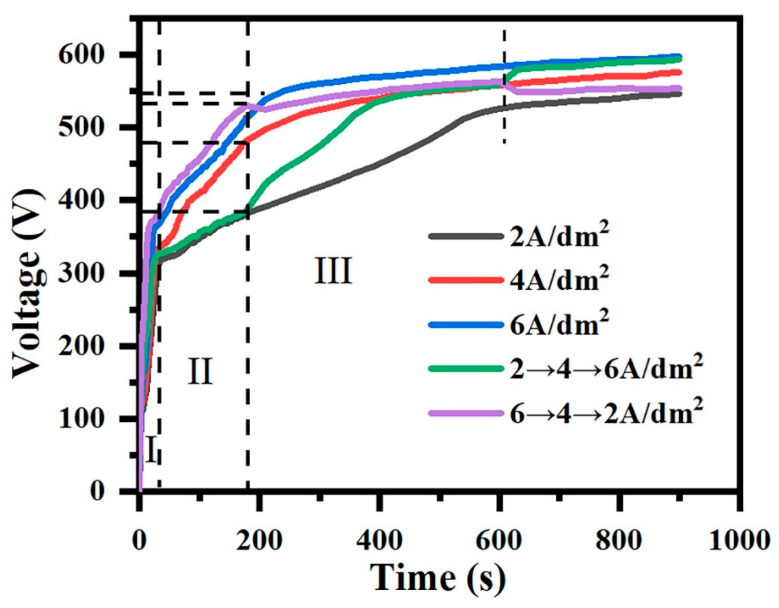
The curves of voltage versus time during the micro-arc oxidation process under constant-current mode (2, 4, 6 A/dm^2^) and gradient-current mode (“246”, “642”). (I: The anodizing stage; II: Spark discharge stage; III: Micro-arc oxidation stage).

Figure 1 shows the curve of the voltage over time in different current output modes. For constant-current modes (2, 4, 6 A/dm^2^), the voltage rise process can be divided into three stages [32]. In stage I, the voltage rises sharply, and the alloy surface elements react chemically in the electrolyte to form an oxide film. This process is called the anodizing stage. Subsequently, in stage II, when the voltage applied to the sample reaches the breakdown voltage, the voltage rise slows down, a white spark discharge appears, and a ceramic layer forms on the surface of the sample; however, at this time, the ceramic layer grows slowly, with low hardness and density. When the voltage exceeds the critical voltage, it enters stage III. The voltage rise slows down further, and the spark becomes larger and brighter and is accompanied by a blast sound; this is the micro-arc oxidation stage.

For gradient-current mode, when the current density is from 2 A/dm^2^ to 4 A/dm^2^ and from 4 A/dm^2^ to 6 A/dm^2^, there are two rising nodes at the voltage with the rise in the current density. Similarly, when the current density drops, a falling node will also appear at the fall. The termination voltage in the current density drop mode is lower than that in the current density rise mode.

**Figure 2:**
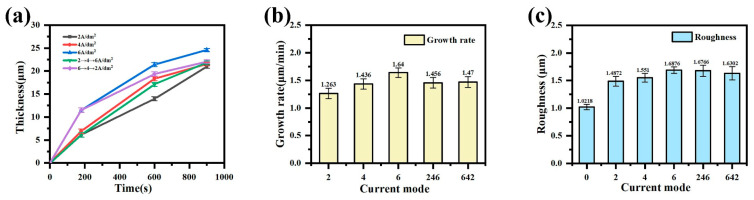
The (**a**) thickness, (**b**) growth rate, and (**c**) roughness of the MAO coating in different current output modes.

Figure 2a shows the curve of the coating thickness over time under different current output modes, and Figure 2b shows the overall growth rate of the coating under different current output modes. In the constant-current mode, the coating thickness increases with the treatment time, and the coating thickness also increases with the increase in current density. The step slows down and increases with the increase in current density. In the gradient-current mode, the growth rate of the coating gradually increases with time in the “246” mode. In contrast, the growth rate of the coating gradually decreases with time in the “642” mode, but the overall growth rate of the coating is slightly higher in the “642” mode.

Figure 2c shows the roughness of the coating in different current output modes. During the micro-arc oxidation process, as the coating thickness increases, the roughness also increases. This is because, in the later stage of micro-arc oxidation, the discharge energy of the micro-zone increases and the reaction is violent. The oxide is melted instantly at high temperatures. Then, the electrolyte causes it to cool and solidify quickly, and some of the molten substances can even melt again before they completely solidify. This repeatedly causes it to accumulate at the discharge port to form a convex port. At the same time, the aperture of the discharge channel increases, and the accumulation range becomes increasingly large, showing a gully shape, resulting in the surface becoming increasingly rough. As such, the roughness increases with the increase in current density.

The programmed current modes regulate the discharge energy through current density modulation. The “246” mode demonstrates an ascending energy intensity, while the “642” mode exhibits reverse energy gradation. Both modes yield lower surface roughness than the constant-current 6 A/dm^2^ treatment, albeit higher than in the 2 and 4 A/dm^2^ modes. In the “246” mode, initial low-energy discharges (2–4 A/dm^2^ phase) generate a smooth coating surface (Ra = 0.721 μm). Subsequent high-energy discharges (6 A/dm^2^ phase) induce localized microstructural defects, but the pre-formed dense layer effectively restricts overall roughness escalation. Conversely, the “642” mode produces higher initial roughness (Ra = 1.272 μm) due to intensive discharges during the 6 A/dm^2^ phase. Progressive current reduction in later stages (4 → 2 A/dm^2^) promotes the molten oxide filling of pre-existing pores, achieving partial surface planarization.

A comprehensive evaluation considering the coating thickness, surface roughness, and growth rate confirms the “642” mode’s optimal performance, achieving balanced structural integrity and deposition efficiency.

The growth of micro-arc oxidation coatings is a cycle in which discharge causes the coating to undergo “breakdown–melt–deposition”. The discharge energy is crucial to the growth of micro-arc oxidation coatings [5]. Here, the energy consumption during the micro-arc oxidation process is calculated using Formula (1):

(1) W=P⋅td⋅S=UI⋅td⋅S=I∫U(t)dtd⋅S

*W* represents the energy consumption (kW·h/(m^2^·μm)), *P* represents the effective power (w) in the micro-arc oxidation process, *U* is the effective voltage (V), *I* is the effective current (A), *t* is the micro-arc oxidation time (h), d is the micro-arc oxidation coating film thickness (μm), and *S* is the sample surface area (m^2^). The energy consumption results in different current modes are shown in Table 1.

Under constant-current conditions, the energy consumption increases proportionally with current density elevations. Each 2 A/dm^2^ increment in the current density corresponds to approximately 40% higher energy expenditure. This phenomenon arises from two interdependent mechanisms: (1) increased breakdown voltage requirements accompanying coating thickness growth necessitate greater discharge energy, and (2) excessive current densities induce repeated dielectric breakdowns, diverting energy towards electrolyte ionization and plasma channel formation rather than oxide deposition. Notably, approximately 35–45% of the input energy dissipates as thermal losses through electrolyte convection, with only 18–22% contributing to oxide melting/redeposition processes. This thermal conversion imbalance explains the accelerated electrolyte temperature rise observed at higher current densities.

The programmed current mode optimizes energy utilization through staged current regulation. The “642” mode demonstrates 0.5% lower energy consumption than the “246” mode at equivalent coating thicknesses. Initial high-current phases (6 A/dm^2^) accelerate initial film formation, reducing non-productive energy consumption during early oxidation. Subsequent current reduction phases minimize the thermal conversion ratios, effectively curbing energy dissipation.

Based on Figure 1, the “642” mode can achieve phased step reduction, which can reduce the ineffective energy consumption of the coating being repeatedly broken down and avoid excessive discharge and energy loss caused by a continuous high current density. Although the constant-current mode has minimum energy consumption at 2 A/dm^2^, its film formation rate makes it unsuitable for actual production; in summary, the gradient-current mode is conducive to reducing the energy consumption, and the “642” mode’s effect is superior.

**Figure 3:**
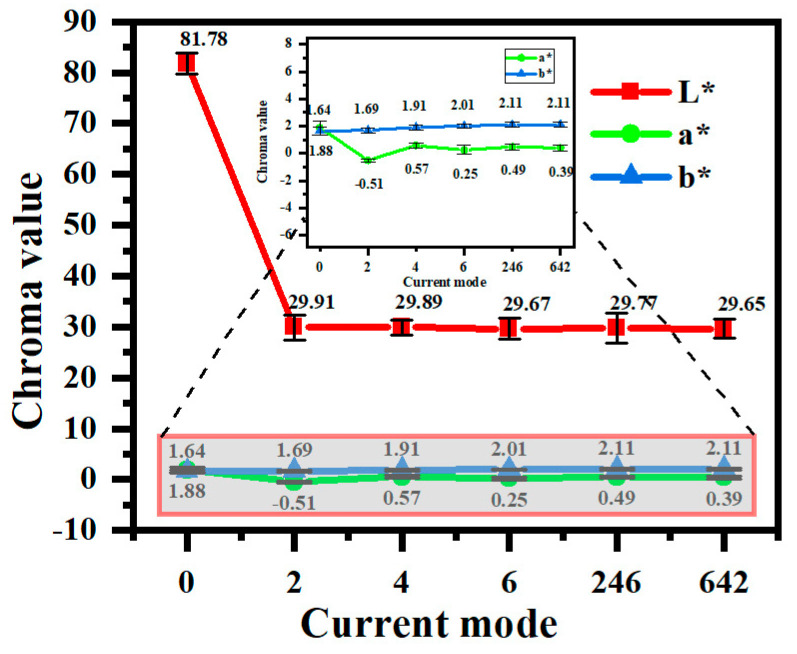
Color values of micro-arc oxidation coatings under different current output modes.

Figure 3 shows the color values of the coating in different current output modes. From the analysis of the L*a*b* value, it can be seen that, in the constant-current mode, the L* value of the micro-arc oxidation coating decreases with the increase in current density, i.e., the blackness of the micro-arc oxidation coating deepens. Elevated current densities induce intensified discharges that promote the reduction of VO_3_^−^ to V^3+^, thereby increasing the V_2_O_3_ content in the coatings. As demonstrated in Table 2, V_2_O_3_ exhibits characteristic black coloration, directly contributing to enhanced coating darkness. Furthermore, an increased current density correlates with a greater coating thickness and surface roughness. This combination amplifies light absorption through multiple internal reflections and surface scattering effects, synergistically intensifying the perceived blackness.

Under programmed current modes, the “642” configuration achieves lower L* values compared to both the “246” and constant-current modes. Initial high-current phases (6 A/dm^2^) accelerate electrolyte breakdown, facilitating NH_4_VO_3_’s dissociation into VO_3_^−^, which undergoes sequential reduction to V^4+^ and V^3+^ during discharge events. Subsequent current reduction further promotes VO_3_^−^‘s reduction to V^3+^, progressively elevating the V_2_O_3_ content. The final low-current phases minimize the discharge energy, effectively suppressing electrolyte decomposition while preventing the oxidation reversal of V^4+^/V^3+^ to V^5+^, thereby stabilizing vanadium’s oxidation state and ensuring chromatic uniformity. The main reactions of the above process are as follows:

(2) $${NH}_{4}{VO}_{3}\to {{NH}_{4}}^{+}+{{VO}_{3}}^{-}$$

(3) $${{VO}_{3}}^{-}+H_{2}O+e^{-}\to V_{2}O_{3}+{OH}^{-}$$

(4) $${{VO}_{3}}^{-}+H^{+}+e^{-}\to {VO}_{2}+H_{2}O$$

In terms of constant-current or gradient-current mode, from the perspective of preparing black coatings, the L value of the coating is lower than the production requirement, and the gradient mode “642” provides the best effect.

**Figure 4:**
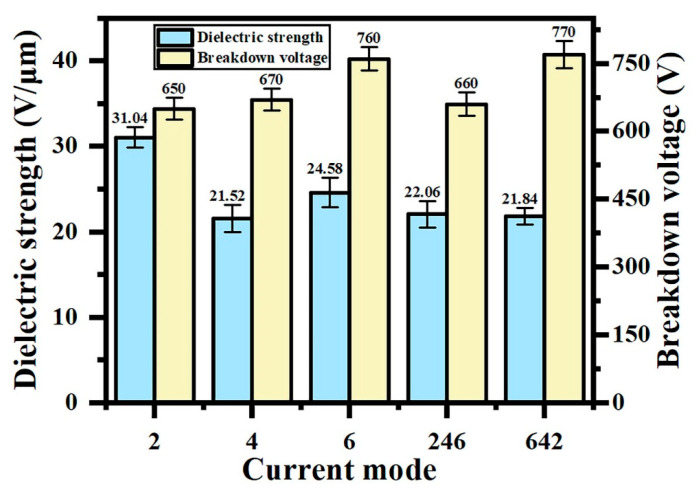
Dielectric strength and breakdown voltage of micro-arc oxidation coatings under different current output modes.

Figure 4 shows the dielectric strength and breakdown voltage of the coating in different current output modes. In constant-current mode, the breakdown voltage of the coating is positively correlated with the thickness because it extends the breakdown path and requires more energy for insulation breakdown. The “642” programmed current mode demonstrates a superior breakdown voltage compared to both the “246” and constant-current modes. Initial high-current discharges (6 A/dm^2^ phase) promote rapid film formation, where the concentrated energy input enhances the arc initiation efficiency, effectively reducing the through-pore density. Intermediate current reduction (4 A/dm^2^ phase) mitigates localized abnormal discharges caused by excessive energy, improving the breakdown voltage uniformity (coefficient of variation < 8%). Final low-current operation (2 A/dm^2^ phase) facilitates the partial sealing of macropores (5–12 μm diameter) and crack repair through oxide redeposition, thereby reducing the local conductivity.

To eliminate thickness-dependent effects, the dielectric strength (breakdown voltage to thickness ratio) was adopted for insulation assessment. As quantitatively demonstrated in Figure 4, the “642” mode achieves the maximum dielectric strength (35.25 V/μm), confirming the optimal insulation performance. This enhancement directly correlates with improved coating uniformity and densification achieved through staged energy regulation.

### 3.2. Effects of Current Output Mode on Microstructure of Micro-Arc Oxidation Coating

**Figure 5:**
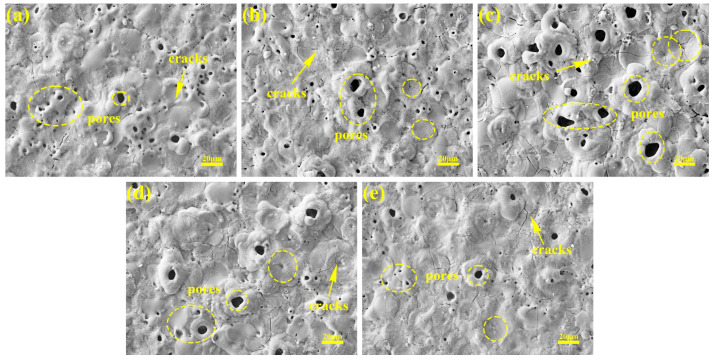
Surface SEM micrographs of micro-arc oxidation coatings in different current modes: (**a**) 2 A/dm^2^, (**b**) 4 A/dm^2^, (**c**) 6 A/dm^2^, (**d**) “246”, (**e**) “642”.

Figure 5 shows the surface morphology of the micro-arc oxidation coating prepared in different current output modes. The coating surface comprises dispersed micropores and irregularly distributed sintered molten oxide grains, where micropores correspond to discharge channels formed during micro-arc oxidation, while grains represent oxide deposits ejected from these channels under plasma conditions. As evidenced in Figure 5a–c, constant-current operation exhibits current-density-dependent structural evolution: elevated current densities (6 A/dm^2^) intensify plasma discharges, generating enlarged discharge channels that collectively form a honeycomb-like porous architecture. Concurrently, transient high-temperature melting followed by rapid solidification generates micrometer-scale protrusions, consistent with Figure 2c. Sustained thermal exposure under high-current conditions further induces crack propagation through accumulated thermal stresses.

Compared with their constant-current counterparts, the coatings fabricated under the “246” programmed current mode exhibit intermediate pore dimensions between those of the 4 and 6 A/dm^2^ constant-current treatments, yet they demonstrate reduced surface protrusion densities and 23–35% lower crack counts, attributable to the initial low-current phase’s smoothing effect. The “642” mode achieves minimal pore dimensions and crack densities through late-stage current reduction, where the diminished discharge energy enables the partial filling of macropores with molten oxides.

**Figure 6:**
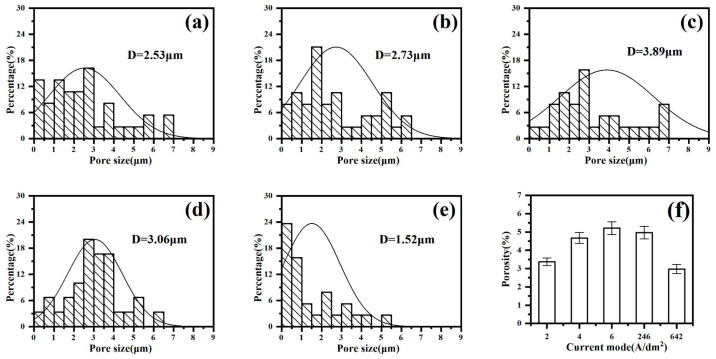
Pore size distribution and porosity of micro-arc oxidation coatings under different current output modes: (**a**) 2 A/dm^2^, (**b**) 4 A/dm^2^, (**c**) 6 A/dm^2^, (**d**) “246”, (**e**) “642”, (**f**) porosity.

To further analyze the coating morphology, the micropore diameter and number of micropores were measured using the ImageJ(ImageJ-win64) software to obtain a histogram of the micropores (Figure 6a–e). The pore size is mainly distributed between 0 and 9 μm. The average pore size of the coating in the “642” mode is the smallest at 1.52 μm. The constant-current mode “6 A/dm^2^” yields the maximum at 3.89 μm. At the same time, the gaps in the morphology obtained via SEM on the coating surface were calculated, and the results are shown in Figure 6f. In constant-current mode, as the current density increases, the porosity of the coating also increases. The coating has the lowest porosity under the “642” mode, at only 2.97%. Along with the smallest average pore diameter in this mode, it can be seen that the coating density is the best.

**Figure 7:**
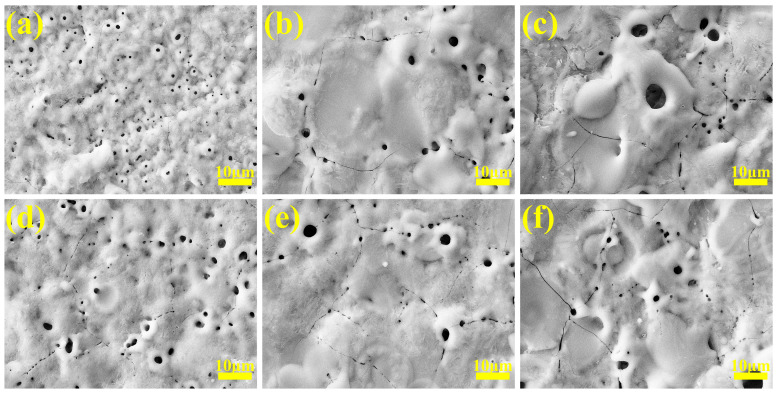
Surface SEM micrographs of micro-arc oxidation coatings at different stages in gradient-current modes: (**a**–**c**) “246”, (**d**–**f**) “642”.

To demonstrate that the micro-arc-oxidized samples prepared in gradient-current mode are superior to those in constant-current mode, Figure 7 shows the surface morphologies of coatings at different stages under a gradient current. Figure 7a shows the surface morphology under the micro-arc oxidation reaction for a period of time at 2 A/dm^2^. Due to the small current density, micro-arc oxidation starts slowly, and there are only tiny sparks on the surface. At this time, the coating film generated is low in thickness and the micropores are very small. After adjusting the current density to 4 A/dm^2^ for a period of reaction, the surface pore size slightly increases and the number of micropores decreases. This is because, as the coating thickness increases, it can be seen from Figure 1 that the current density of 2 A/dm^2^ cannot provide enough energy for the coating to grow. At this time, increasing the current density simply compensates for the lack of energy, the coating continues to grow, and some molten oxides cover the initial holes, resulting in a decrease in the number of holes. We continued to adjust the current density to 6 A/dm^2^ until the reaction was over. It can be seen from Figure 7c that the coating pore size has further increased, the cracks have increased, and the surface particles are protruding. This is because the discharge energy is too large at this time, the discharge channel is expanded, and some oxides even undergo “melting–cooling–melting” and continue to accumulate to form convex holes. Thermal stress caused by instantaneous high temperatures also causes the coating to continue to crack.

Under the programmed current “642” mode, the initial maximum current density (6 A/dm^2^) ensures sufficient arc initiation energy at the substrate surface, enabling rapid plasma ignition with balanced energy distribution per discharge site, thereby promoting uniform coating growth. During subsequent processing at 4 A/dm^2^, reduced discharge energy maintains continuous coating deposition. This stage exhibits pore expansion coupled with partial pore filling via oxide redeposition. In the final stage (2 A/dm^2^), the diminished discharge energy prolongs the oxide melt–resolidification cycle, facilitating progressive micropore filling and pore diameter reduction. Concurrent current reduction alleviates thermal stress accumulation, yielding a decline in crack density compared to high-current phases.

**Figure 8:**
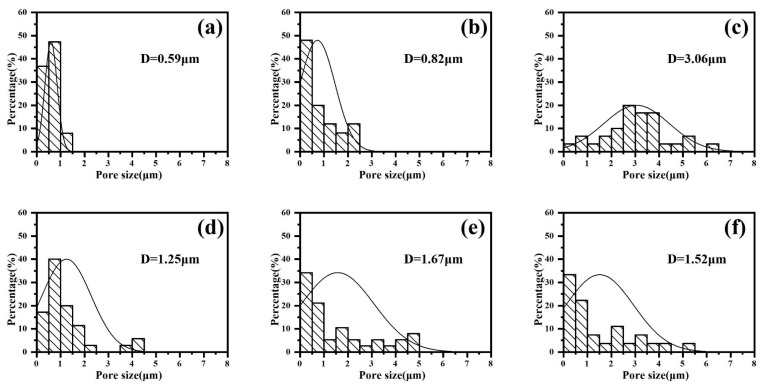
Pore size distribution of micro-arc oxidation coatings at different stages in gradient-current modes: (**a**–**c**) “246”, (**d**–**f**) “642”.

The histogram of the coating pore sizes at different stages in gradient-current mode is shown in Figure 8. It can be seen that, under the gradient mode “246”, the average pore diameter of the coating continues to increase with the adjustment of the current density, while the average pore diameter in the “642” mode decreases after the last adjustment of the current density, i.e., the small current density provides the molten oxide with enough time to repair the coating holes by reducing the discharge energy.

**Figure 9:**
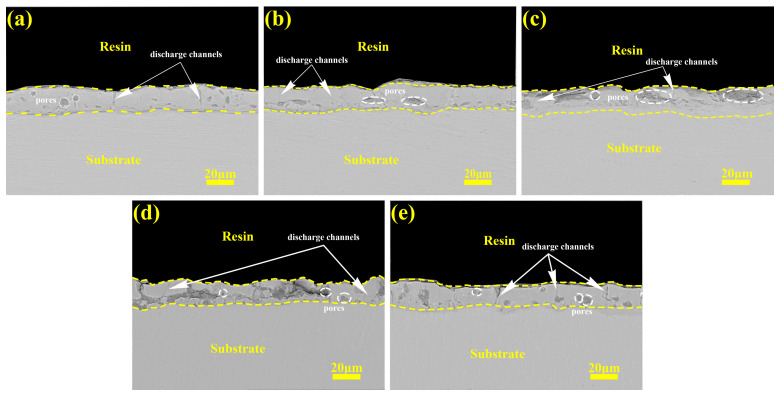
The cross-section micrographs of micro-arc oxidation coatings under different current output modes: (**a**) 2 A/dm^2^, (**b**) 4 A/dm^2^, (**c**) 6 A/dm^2^, (**d**) “246”, (**e**) “642”.

Figure 9 shows the cross-sectional morphology of the prepared micro-arc oxidation coating. As shown in the figure, in constant-current mode, as the current density increases, violent discharge produces more micro-arc discharge channels, and microcracks and honeycomb-like holes form inside the coating. The number of micropores in the coating increases, the discharge is energized, the porosity increases, and the coating becomes looser. Moreover, the surface of the coating becomes increasingly uneven, which is consistent with the results in Figure 2. In gradient-current mode, the later high current of the “246” mode will increase the damage to the coating by discharge, which is shown in the figure, where the cross-section is filled with discharge channels and cracks. The small current in the later stage of the “642” mode will fill and repair the old large discharge channels and cracks.

Three distinct discharge types are generally recognized during micro-arc oxidation: surface-proximal A-type discharges, gas-mediated C-type discharges within coating voids, and through-coating B-type discharges penetrating the coating–substrate interface [33]. A cross-sectional analysis reveals current modulation’s direct influence on the anode sparking characteristics through coating morphological evolution. Under constant-current conditions, elevated current densities induce a progressive voltage escalation, intensifying discharge energies that preferentially promote type B discharges during prolonged processing [34]. This mechanism causes coating–substrate interfacial degradation, with exacerbated damage observed under the “246” mode. The “642” descending-current mode demonstrates voltage reductions at current transitions, effectively suppressing type B occurrences. The dominant A-/C-type discharges in later stages facilitate macropore filling through controlled plasma redeposition. This operational logic determines the current-switching thresholds—timely current adjustments at discharge-type transitions enable dynamic plasma control for optimized coating repair.

### 3.3. Effects of Current Output Modes on Corrosion Resistance of Micro-Arc Oxidation Coatings

**Figure 10:**
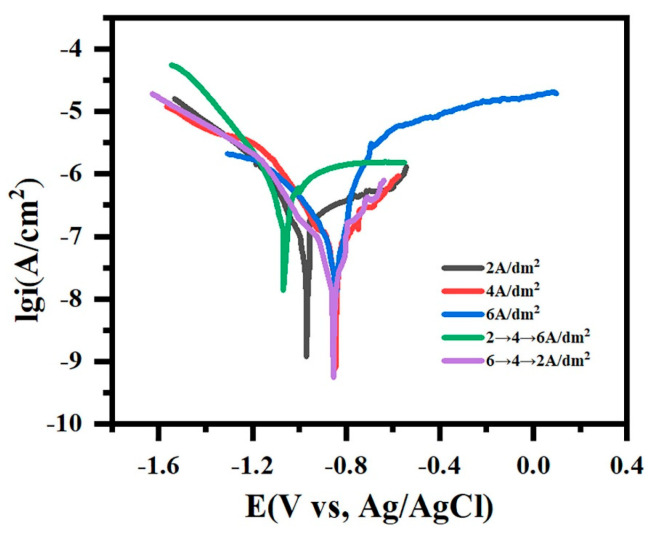
The potentiodynamic polarization curves of micro-arc oxidation coatings under different current output modes immersed in 3.5 wt.% NaCl solution for 60 min.

Figure 10 presents the potentiodynamic polarization curves of micro-arc oxidation coatings under varied current modes in 3.5 wt.% NaCl solution, with the corresponding fitting parameters detailed in Table 3. The corrosion potential (E_corr_) reflects the coating stability, while the corrosion current density (i_corr_) quantifies the degradation kinetics, where elevated E_corr_ and reduced i_corr_ indicate enhanced corrosion resistance [35,36]. The gradient-current “642” mode exhibits the best E_corr_ (−0.837 VSCE) and a minimal i_corr_ (2.55 × 10^−8^ A/cm^2^), confirming its superior barrier properties. The constant-current modes at 4/6 A/dm^2^ demonstrate comparable i_corr_ values (~6.3 × 10^−8^ A/cm^2^) yet suffer from enlarged micropores that accelerate corrosive medium infiltration. The 2 A/dm^2^ mode, despite smaller pores, shows compromised protection due to insufficient coating thicknesses, permitting ionic penetration. The worst-performing “246” mode displays severe corrosion susceptibility (i_corr_ = 2.34 × 10^−7^ A/cm^2^), attributable to the interconnected macroporosity in the cross-sectional morphology that facilitates direct electrolyte–substrate interaction.

Corrosion manifests as chemical and electrochemical forms [37], with pure chemical corrosion being exceptionally rare in practical engineering systems. In electrochemical corrosion processes, the resistivity of oxide constituents governs the charge transport capacity during corrosive reactions—higher resistivity corresponds to slower charge transport and reduced corrosion rates [38,39]. Thus, disregarding the thickness and compactness factors, enhanced coating resistivity directly correlates with improved corrosion resistance. Table 4 lists the resistivity values of various coating oxides. Vanadium oxides exhibit significantly lower resistivity (10^−3^–10^3^ Ω·cm) compared to aluminum (10^14^ Ω·cm) and silicon oxides (10^16^ Ω·cm); the former tend to form microscopic electric pairs within the coating, thereby accelerating its dissolution. The excellent conductive ability of vanadium oxide means that it can form a continuous or local conductive grid in the originally high-resistance aluminum oxide coating, which leads to a decrease in the overall electrical resistance of the coating. The layered structure of vanadium oxide itself also provides migration channels for corrosive media, accelerating the diffusion of corrosive media to the substrate. Furthermore, a discrepancy in the coefficient of thermal expansion exists between the vanadium oxide and the substrate oxide, which is likely to induce substantial residual stresses within the coating. Consequently, this may result in the formation of microcracks, which are the primary conduit for the ingress of corrosive media, indicating vanadium’s detrimental role in creating conductive pathways that accelerate corrosion propagation.

**Figure 11:**
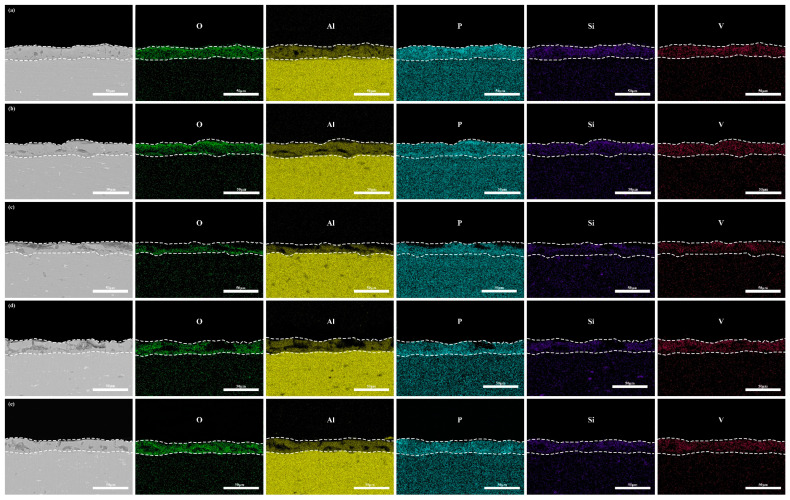
EDS of micro-arc oxidation coating cross-sections under different current modes: (**a**) 2 A/dm^2^, (**b**) 4 A/dm^2^, (**c**) 6 A/dm^2^, (**d**) “246”, (**e**) “642”.

An EDS analysis (Figure 11) reveals uniform vanadium distribution without localized enrichment, consistent with the surface morphology in Figure 5. Cross-sectional quantification (Table 5) demonstrates minimal V content (1.55 wt.%) in the “642” mode, aligning with its optimal polarization behavior. Although the “246” mode contains less V (1.75 wt.%) than the constant-current modes (2.08–2.12 wt.%), excessive discharge channels dominate its inferior corrosion resistance. Coating durability emerges from synergistic interactions among the microstructural features, phase composition, and dimensional characteristics.

## 4. Conclusions

This study is of great significance to the physical protection of aluminum and its alloys, and improves the corrosion resistance of micro-arc oxidation coatings to a certain extent. The influenceof the gradient current output mode on the micro-arc oxidation coating was studied. Since the change of the traditional gradient output mode takes the oxidation time as the node, the influence of the micro-arc oxidation spark discharge on the microstructure, structure and properties of the coating is neglected. Therefore, this study adjusts the spark discharge during the micro-arc oxidation process by gradient output to affect the performance of the coating. The following conclusions are obtained: Compared with the constant current output mode and the gradient increase current output mode, the gradient decrease current output mode (‘642’ mode):

(1) At the same time, the coating has the lowest blackness value and the fastest growth rate under smaller roughness. Energy consumption is also reduced by 31.1%.
(2) Adjusting the current density accelerates arcing in the early stages with a large current, while a small current in the middle and late stages ensures coating growth, reduces discharge energy output and repairs defects such as pores and cracks. This decreases the average pore size of the coating by 1–2 μm and reduces porosity by 40%.
(3) The coating has the best corrosion resistance, the most positive corrosion potential, and the corrosion current density is reduced by an order of magnitude.

## Figures and Tables

**Table 1:** Energy consumption of micro-arc oxidation under different current output modes.

Current Mode	*P* (w)	*t* (h)	*d* (μm)	*S* (m^2^)	*W* (kw·h/(m^2^·μm))
2	227	0.25	18.94	0.0024	1.25
4	513.42	0.25	21.52	0.0024	2.49
6	813.68	0.25	24.58	0.0024	3.45
246	499.49	0.25	21.84	0.0024	2.38
642	501.98	0.25	22.06	0.0024	2.37

**Table 2:** The colors of various oxides of vanadium.

Oxide	Color	Oxide	Color
VO	gray-black	V_2_O_3_	black
VO_2_	dark blue	V_2_O_5_	buff

**Table 3:** The results of the potentiodynamic polarization tests.

Current Mode	E_corr_ (mV vs. Ag/AgCl)	I_corr_ (A/cm^2^)
2	−977	1.62 × 10^−7^
4	−846	6.36 × 10^−8^
6	−845	5.36 × 10^−8^
246	−1059	2.34 × 10^−7^
642	−837	2.55 × 10^−8^

**Table 4:** The conductivity of oxides (25 °C).

Oxide	Conductivity /Ω·cm	Oxide	Conductivity /Ω·cm
SiO_2_	10^16^	VO	10^−3^
Al_2_O_3_	10^14^	V_2_O_3_	10^−3^
VO_2_	10^2^	V_2_O_5_	10^2^–10^3^

**Table 5:** EDS composition analysis of cross-section compositions of micro-arc oxidation coatings.

Sample	Percentage of Element Content (wt. %)				
	Al	O	Si	P	V
a	80.83	7.92	1.54	7.63	2.08
b	82.31	6.54	1.46	7.62	2.07
c	83.37	5.99	0.87	7.65	2.12
d	85.10	7.52	1.37	4.25	1.75
e	82.67	9.27	1.36	4.95	1.55

## Data Availability

The original contributions presented in this study are included in the article. Further inquiries can be directed to the corresponding author.
